# Synthetic Lethal Activity of Benzophenanthridine Alkaloids From *Zanthoxylum coco* Against BRCA1-Deficient Cancer Cells

**DOI:** 10.3389/fphar.2020.593845

**Published:** 2020-12-03

**Authors:** Iris A. García, Maria Florencia Pansa, Adriana Del Valle Pacciaroni, Manuela E. García, Maria Laura Gonzalez, Juan Carlos Oberti, José Luís Bocco, Maria Cecilia Carpinella, Gloria E. Barboza, Viviana E. Nicotra, Gastón Soria

**Affiliations:** ^1^ Centro de Investigaciones en Bioquímica Clínica e Inmunología, CIBICI-CONICET, Córdoba, Argentina; ^2^ Departamento de Bioquímica Clínica, Facultad de Ciencias Químicas, Universidad Nacional de Córdoba, Córdoba, Argentina; ^3^ Instituto Multidisciplinario de Biología Vegetal, IMBIV-CONICET, Córdoba, Argentina; ^4^ Departamento de Química Orgánica, Facultad de Ciencias Químicas, Universidad Nacional de Córdoba, Córdoba, Argentina; ^5^ Instituto de Investigaciones en Recursos Naturales y Sustentabilidad Jose Sanchez Labrador S.J., IRNASUS-CONICET, Córdoba, Argentina

**Keywords:** natural products, *Zanthoxylum coco*, oxynitidine, nitidine, drug discovery, BRCA1, synthetic lethality

## Abstract

Several plants from South America show strong antitumoral properties based on anti-proliferative and/or pro-apoptotic activities. In this work we aimed to identify selective cytotoxic compounds that target BRCA1-deficient cancer cells by Synthetic Lethality (SL) induction. Using a high-throughput screening technology developed in our laboratory, we analyzed a collection of extracts from 46 native plant species from Argentina using a wide dose-response scheme. A highly selective SL-induction capacity was found in an alkaloidal extract from *Zanthoxylum coco* (Fam. Rutaceae). Bio-guided fractionation coupled to HPLC led to the identification of active benzophenanthridine alkaloids. The most potent SL activity was found with the compound oxynitidine, which showed a remarkably low relative abundance in the active fractions. Further validation experiments were performed using the commercially available and closely related analog nitidine, which showed SL-induction activity against various BRCA1-deficient cell lines with different genetic backgrounds, even in the nanomolar range. Exploration of the underlying mechanism of action using BRCA1-KO cells revealed AKT and topoisomerases as the potential targets responsible of nitidine-triggered SL-induction. Taken together, our findings expose an unforeseen therapeutic activity of alkaloids from *Zanthoxylum*-spp. that position them as novel lead molecules for drug discovery.

## Introduction

BRCA1 is highly characterized as a tumor suppressor gene that was originally identified in hereditary breast and ovarian cancers (King et al., 2012; Takaoka and Miki, 2018). Nevertheless, there is increasing evidence indicating that alterations in BRCA1, such as mutations or epigenetic downregulation, are also recurrently found in sporadic cancers, being the genome instability triggered by BRCA1 deficiency the driving force of tumorigenesis (Fackenthal and Olopade, 2007; Venkitaraman, 2014). Moreover, several recent clinical studies have shown that BRCA1-deficient phenotypes are found with high prevalence not only in breast and ovarian cancers, but also in pancreatic, prostatic, and other types of cancers (Lord and Ashworth, 2016).

A strategy that takes advantage of the genetic differences of cancer cells to achieve their selective death is known as synthetic lethality (SL), which consists in the simultaneous inactivation of compensatory or redundant molecular pathways (Canaani, 2013; Nijman and Friend, 2013; Ryan et al., 2018). In this sense, in a BRCA1-deficient context, the inhibition of specific molecular targets that compensate BRCA1 functions could trigger SL. A successful example of SL-induction to target BRCA-deficient cancers is the development and approval of PARP inhibitors (PARPi) (Kohn et al., 2017; Lord and Ashworth, 2017), which have recently reached different types of clinical settings (Golan et al., 2019; González-Martín et al., 2019; Saleh and Copur, 2019) (https://www.fda.gov/drugs/drug-approvals-and-databases/fda-approves-niraparib-first-line-maintenance-advanced-ovarian-cancer). While PARPi exploit the convergent roles of BRCA1, BRCA2 and other genes involved in Homologous Recombination (HR) DNA repair, BRCA1 also functions in many other cellular processes that set the ground for novel SL-induction scenarios (Venkitaraman, 2014; Sharma et al., 2018; Simhadri et al., 2019). Moreover, given that many types of resistance mechanisms to PARPi are already being described (D’Andrea, 2018; Noordermeer and van Attikum, 2019), there is a major interest in developing alternative SL strategies to target BRCA1 deficiency from different angles.

From an historical perspective, natural products contributed to the origin of medicine. They provide a great diversity of metabolites and present structural features that synthetic compounds do not have (Henkel et al., 1999; Rodrigues et al., 2016; Newman and Cragg, 2020), so it is not surprising that many of the available drugs derive from plants and microorganisms (Harvey, 2008; Thomford et al., 2018). The enormous impact of natural products in oncology drug discovery cannot be overestimated. Nearly 50% of antineoplastic agents or drugs in clinical trials are either natural products or derived compounds (Bindseil et al., 2001; Schmidt et al., 2007; Trendowski, 2015; Dutta et al., 2019). Natural products can be obtained from varied sources, such as plants, algae and fungi. This is particularly striking in Argentina, where there is a huge biodiversity in its flora and countless unexplored plant species.

Using a high-throughput screening technology developed in our Lab (Carbajosa et al., 2019; Villafañez et al., 2019), we analyzed a panel of 46 extracts obtained from native and naturalized plant species from Argentina. An alkaloidal extract from *Zanthoxylum coco* Gillies ex Hook. f. & Arn. [*Fagara coco* (Gillies ex Hook. f. & Arn.) Engl.] (Fam. Rutaceae), presented the most selective SL-induction activity, being oxynitidine the most active compound. These findings unveil a novel therapeutic activity of alkaloids from *Zanthoxylum*-spp., which will be of interest as lead molecules for future drug discovery projects.

## Materials and Methods

### General Experimental Procedures

NMR spectra were recorded on a Bruker AVANCE II AV-400 instrument operating at 400.13 MHz for ^1^H and 100.63 MHz for ^13^C, while 2D spectra (COSY, HSQC, HMBC, and NOESY) were obtained using standard Bruker software.

Chromatographic separations were performed by column chromatography on silica gel 60 (0.063–0.200 mm), preparative TLC on silica gel 60 F_254_ (0.2 mm thick) plates, and HPLC. Preparative TLC separations were performed under the following conditions: i) the amount of sample applied was approximately 15 mg for 20 cm plate; ii) the bands were visualized using ultraviolet light and Dragendorff reagent; iii) compounds were eluted from the silica using hexane:EtOAc (8:2).

HPLC separations were achieved on a Waters HPLC set provided with binary pump (Waters 1525), Agilent column (ZORBAX Eclipse XDB-C18, 9.4 × 250 mm, 5-μm), UV-Vis detector with diode array (Waters 2998); a Rheodyne manual injector valve, with 80 µl sample loop. Chromatograms were processed with Empower software.

### Plant Materials and Extracts Preparation

The plants were collected from December to March at time of flowering. Botanical identification and authentication were carried out by the botanists G. Ruiz and G. Barboza. Voucher specimens have been deposited in the “Marcelino Sayago” Herbarium, Catholic University of Córdoba, in the Herbarium from the Museum of Natural Sciences, National University of Salta and in the Botanical Museum of Córdoba, National University of Córdoba (see Supplementary Table S1, Supplementary Material, which includes the coordinates of collections sites). The crude extracts were obtained by maceration with ethanol or methanol and prepared by exhaustive solvent removal.

*Zanthoxylum coco* was collected in Río Ceballos, Departamento Colón, Córdoba, Argentina (31°09′00′S, 64°18′00″W). Fresh fruits (1,800 g) were minced and extracted with MeOH (3 × 1 L). The solvent was then evaporated at reduced pressure. The total residue was diluted with (500 ml, 10%) aqueous HCl solution. Diatomaceous Earth was added, and the homogenate was placed at 2°C for 12 h. Afterwards, the aqueous phase was vacuum filtrated. The resulting fraction was partitioned in CH_2_Cl_2_ (3 × 200 ml). The pH of the aqueous acidic fraction was adjusted to 9 with NH_4_OH and extracted with CH_2_Cl_2_ (6 × 150 ml). Organic extract was dried over anhydrous Na_2_SO_4_, filtered, and evaporated to dryness at reduced pressure to obtain 1.8 g of alkaloidal extract.

### Bioguided Isolation of the Active Principle in the Alkaloidal Extract From *Zanthoxylum coco*

The alkaloidal extract was fractionated initially by silica gel 60G chromatography. Elution with CH_2_Cl_2_:MeOH mixtures of increasing polarity (100:0–80:20) afforded seven pools. The bioactive fractions pools (III and IV, 750 mg) were subjected to CC with hexane-EtOAc mixtures of increasing polarity (100:0–100:0) yielding five pools. Fractions 8–10 (pool II, 19 mg) was further purified by preparative TLC with hexane:EtOAc (80:20) to give dihydrochelerythrine (7.6 mg). The fractions 11–28 (pools III and IV, 130 mg) were applied to a silica gel 60 G column using hexane:EtOAc mixtures of increasing polarity (100:0–100:0) to afford β-fagarine (9 mg), dictanmine (0.7 mg), and an impure fraction (30 mg), with the latter being further analyzed by high performance liquid chromatography (HPLC). The elution system was composed by phase A: methanol: water [65 :35 (v:v)], phase B: methanol : water [80 :20 (v:v)], and phase C: methanol : water [87 :13 (v:v)]. The HPLC allowed to obtain (in order of elution): 3-(1,2-dihydroxyethyl)-4-methoxy-2(1H)-quinolinone (1.9 mg), haplopine (1.1 mg), β-fagarine (14.5 mg), arnottianamide (0.7 mg), 8-demethyloxychelerythrine (1.3 mg), oxynitidine (2.3 mg), and oxychelerythrine (1.1 mg).

### DNA Constructs and Short Hairping RNA

iRFP-C1 was a gift from Michael Davidson and Vladislav Verkhusha (National Magnetic High Field Laboratory, Tallahassee, FL; Addgene plasmid #54786; Filonov 2012); shBRCA1 (TRCN0000010305, Sigma-Aldrich) was cloned into pLKO.1-TRC vector through EcoRI and AgeI restriction sites; and shSCR-pLKO.1 was described previously (Trucco et al., 2014).

### Cell Culture

HCT116^p21−/−^ cells were kindly provided by Bert Vogelstein (Johns Hopkins Medicine, Baltimore, MD). All the remaining cells used in this study were obtained from ATCC. Cells were cultured in Dulbecco’s modified Eagle’s medium (DMEM; Thermo Fisher Scientific Inc.) supplemented with 10% fetal bovine serum (FBS; GIBCO) and 1% penicillin/streptomycin (Thermo Fisher Scientific Inc.). Control for *Mycoplasma* contamination was performed periodically with a PCR-based method with internal loading control. HCT116^p21−/−^ BRCA-deficient cells (and control cells) were generated as described previously (Carbajosa et al., 2019). Each cell line co-expresses a different fluorescent protein: shSCR (CFP) and shBRCA1 (iRFP). The entire screening phase was performed with cells that did not exceed five passages after transduction to avoid the positive selection of cells with higher BRCA levels. Cell lines were used for experimentation for no more than 20 passages from the main frozen stock. Nitidine was obtained from Sigma-Aldrich (≥97%), diluted in DMSO (Carlo Erba) and used at the indicated concentrations.

### Cell Survival Analysis

Equal numbers of cells were co-cultured or separately plated in 96 MW plates, and increasing doses of extracts/compounds were tested. Six days post-treatment, total cell number of each cell population was counted using automated flow cytometry with an autosampler (Attune NxT, Thermo Fisher Scientific). The relative survival of each cell population in comparison with the untreated controls was determined to calculate SL induction by the different treatments. The PARP inhibitor Olaparib (0.1 μM) was used as positive control of the SL induction.

### Protein Analysis

Western blot analysis was carried out essentially as previously described in (Villafañez et al., 2019). The detection and quantification were performed with Odyssey CLX System (LI-COR Biosciences) using the proprietary Image Studio Software. The following primary antibodies were used: α-phospho-Akt (Ser473; Cell Signaling Technology, Cat# 9271); anti-β-Actin was from (Sigma-Aldrich, Cat# A2228). As secondary antibodies we used: goat α-mouse IRDye 680RD (LI-COR Biosciences, Cat# P/N 925-68070) and goat α-rabbit IRDye 800CW (LI-COR Biosciences, Cat# P/N 925-32211).

### Statistical Analysis

All experiments were executed by duplicate or triplicate. Graphs and statistical analysis were performed using GraphPad Prism 5.0 (GraphPad Software), applying two-sided Student’s *t*-test and ANOVA test as appropriate. Bars represent the mean value ± SD Other calculations were performed using Microsoft Excel 2007.

## Results

### Synthetic Lethal Screen Using a Collection of Extracts From Argentinean Native or Naturalized Plants

**FIGURE 1:**
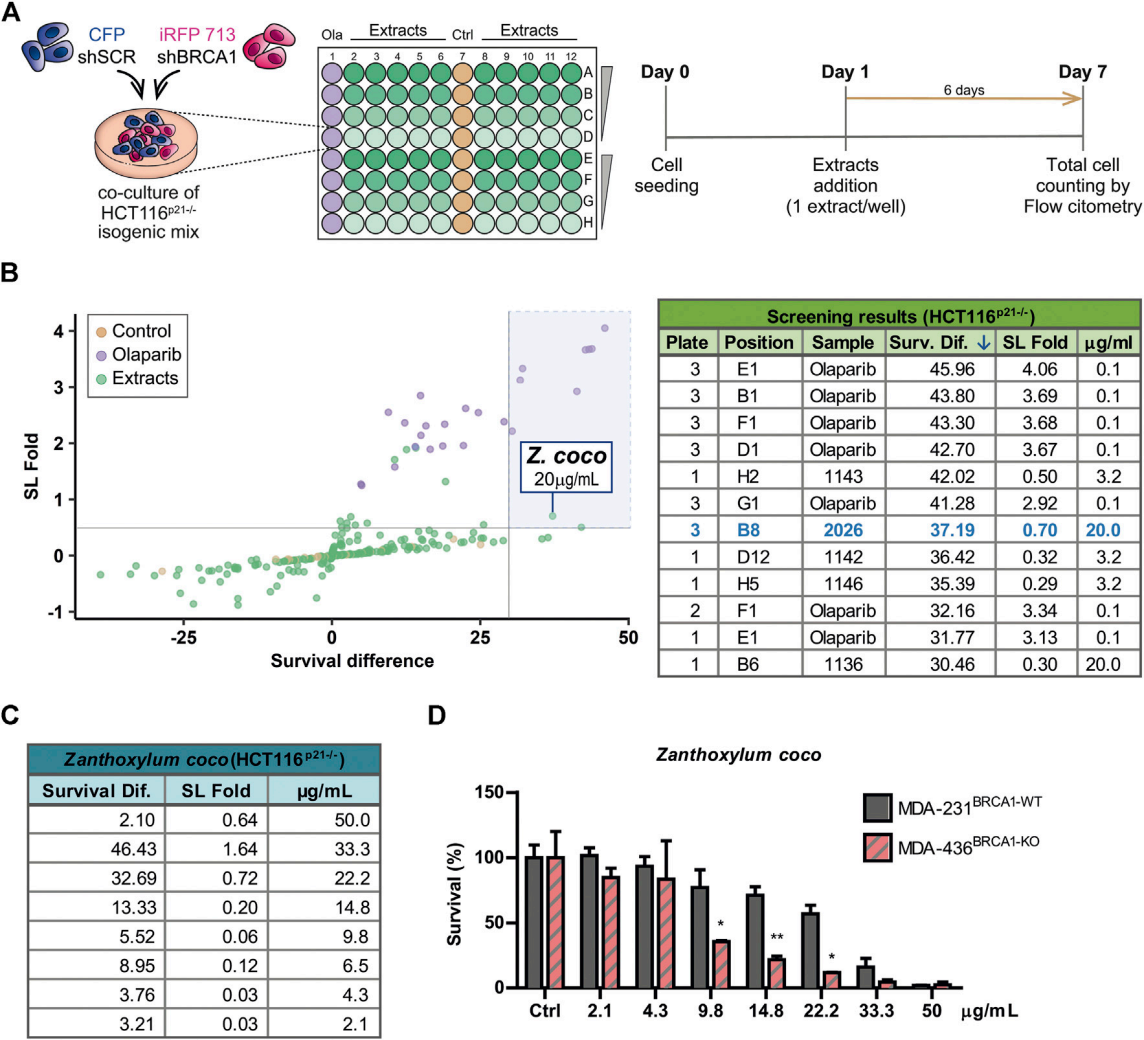
Phenotypic screening reveals that alkaloidal extract of *Zanthoxylum coco* induces synthetic lethality in the BRCA1-deficient population. **(A)** Experimental layout and detailed protocol used to assess synthetic lethality (SL) induction using a co-culture method of BRCA-proficient (shSCR) and BRCA1-deficient (shBRCA1) isogenic HCT116^p21−/−^ cell lines, generated by lentiviral transduction of the corresponding shRNA. Each cell line co-expresses a different fluorescent protein: shSCR (CFP) and shBRCA1 (iRFP). Equal numbers of isogenic cells were then plated in 96 MW plates and treated with increasing doses (3.2, 8, 20, and 50 μg/ml) of extracts from native/naturalized plant species of Argentina. Six days post-treatment, the co-cultured population was counted and categorized by the differential expression of fluorescent proteins using automated flow cytometry with an autosampler. The total cell number of each population/well was determined by an acoustic flow cytometer. PARP inhibitor Olaparib was used as positive control of SL-induction in each screening plate at 0.1 µM. **(B)** Screening results. The graph shows the fold of SL induction and the survival difference after the treatment. Alkaloidal extract from *Zanthoxylum coco* was identified as hit using the criteria of more than 3 SDs in any of the variables. The table is part of Supplementary Table S2 and shows top five hits ordered according the survival difference parameter. **(C)** Results obtained in dose-response experiments in HCT116^p21−/−^ cell lines testing the *Z. coco* extract in a dose range close to that identified in the screening. The SL fold and the survival difference parameters are detailed. **(D)** Validation of the SL response to *Z. coco* extract using nonisogenic MDA-MB pair of breast cancer cell lines in monoculture experiments: MDA-MB-231^BRCA1-WT^ vs. MDA-MB-436^BRCA1-KO^. Statistical analysis was performed using two-way ANOVA with Bonferroni post-test (**, *p* ≤ 0.01; *, *p* ≤ 0.05).

To identify novel SL interactions in BRCA1 deficient contexts, we used an adaptation of a phenotypic screening platform developed in our Lab (Carbajosa et al., 2019; Villafañez et al., 2019) to evaluate a collection of plant extracts. Such collection comprises extracts from 46 native or naturalized plant species from Argentina. The plants belong to various families such as Asteraceae, Bignoniaceae, Celastraceae, Solanaceae, Rutaceae, among other (Supplementary Table S1). We achieved BRCA1 downregulation using short hairping RNAs (shRNAs). In our phenotypic screening platform, two HCT116-derived cell lines expressing shRNAs [shSCR (non-targeting scramble control) and shBRCA1] tagged different fluorescent proteins (CFP and iRFP, respectively) were co-cultured for 6 days in the presence of the evaluated extracts at 2.5-fold serial dilutions: 50, 20, 8 and 3.2 μg/ml (Figure 1 A). The final readout was the total cell number and proportion of each population in each well, which was determined by an acoustic flow cytometer. For screening purposes, hits were defined by a variation greater than 3 SD on two values derived from the differential impact of the extracts on the BRCA1-deficient vs. BRCA1-proficient populations: 1) SL induction fold: derived from the ratio of the proportion of BRCA1-proficient/BRCA1-deficient cells in each well and 2) survival difference: derived from the subtraction of the survival of the BRCA1-deficient from the BRCA1-proficient population. An optimal dose of the PARPi Olaparib (0.1 µM) was used as a positive control of SL-induction (Figure 1; Supplementary Figure S1A).

A careful analysis of the screening results (Figure 1B; Supplementary Table S2), considering both the synthetic lethal fold and survival difference of the hits, focused our attention on the alkaloidal extract of *Zanthoxylum coco* (#2026). Following this initial finding, different *Z. coco* extracts from leaves and fruits were tested, being the alkaloidal extract obtained from fruits the most active one (Supplementary Figure S1B). Such extract showed potent SL-induction, but more importantly, it presented activity within a concentration range where cytotoxicity was evident almost exclusively in the BRCA1-deficient population. This phenotype was observed using different BRCA1-deficient cellular models (Figures 1C,D). Noteworthy, at higher concentrations, the SL-induction capacity of the extract was lost due to the increased unspecific toxicity on the BRCA1-proficient cell line, as it is also observed for the PARPi Olaparib (Supplementary Figure S1A).

### Bio-Guided Fractionation Coupled to High Performance Liquid Chromatography Led to the Identification of Active Furoquinoline and Benzophenanthridine Alkaloids

**FIGURE 2:**
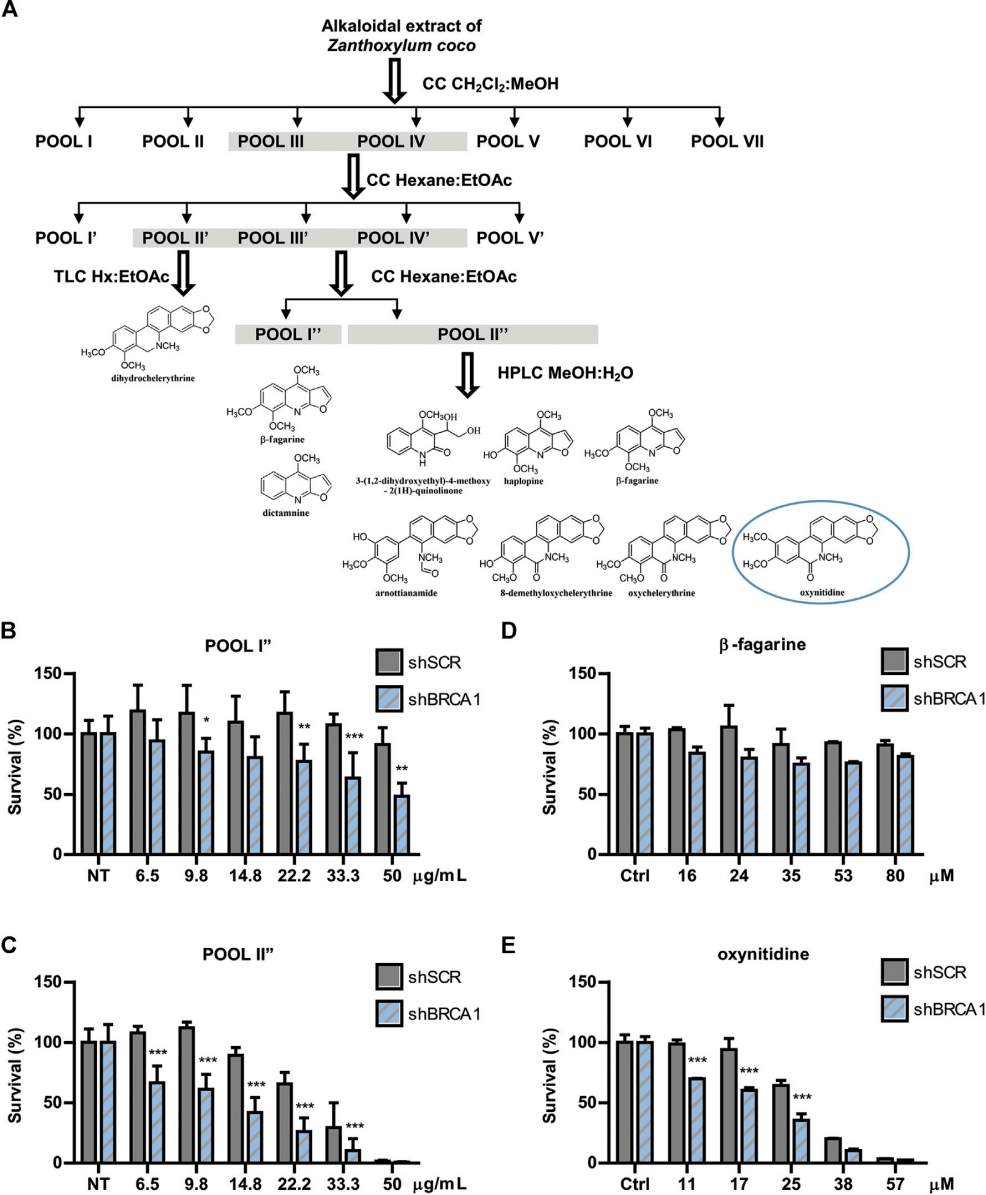
Bioguided separation of the alkaloidal extract of *Zanthoxylum coco* to isolate and identify the active principle responsible for synthetic lethality activity. **(A)** Schematic representation of Bio-guided fractionation coupled to HPLC that led to the isolation and identification of 5 benzophenanthridine alkaloids: dihydrochelerythrine, oxychelerythrine, 8-demethyloxychelerythrine, arnottianamine, and oxynitidine) and 4 furoquinoline alkaloids: (β-fagarine, dictamnine, haplopine, and 3-(1,2-dihydroxyethyl)-4-methoxy-2(1H)-quinolinone). Dose response experiment using POOL I″ **(B)** and POOL II″ **(C)**, showing the survival difference between BRCA1-deficient and proficient cells after 6 days of treatment. Dose response experiment using isolated β-fagarine **(D)** and oxynitidine **(E)** from POOL I″ and POOL II″, respectively, showing the survival difference between BRCA1-deficient and proficient cells after 6 days of treatment. Statistical analysis was performed using two-way ANOVA with Bonferroni post-test (***, *p* ≤ 0.001; **, *p* ≤ 0.01; *, *p* ≤ 0.05).

The active extract from *Z. coco* was subjected to bio-guided fractionation as depicted in Figure 2A. Two fractions exhibited the most robust SL-induction activity, being POOL I″ less potent than POOL II″ (Figures 2B,C, respectively). POOL I″ contained mostly β-fagarine (≥95%), yet this alkaloid presented vestigial SL-induction in comparison to POOL I″ and POOL II″ (Figure 2D). This led us to hypothesize that SL-induction was triggered by a minority compound or a synergy between minority compounds. Therefore, POOL II″ was submitted to reverse-phase HPLC (Figure 2A) to further isolate the active(s) principle(s) responsible for the selective cytotoxicity. From POOL II″, seven alkaloids were isolated, identified and tested for SL-induction. Intriguingly, most of the alkaloids were inactive (Supplementary Figure S2) and the minority compound oxynitidine (<5%), presented the strongest SL-inducing activity (Figure 2E). In summary, *Z. coco* active extract fractionation led to the isolation of five benzophenanthridine alkaloids (dihydrochelerythrine, oxychelerythrine, 8-demethyloxychelerythrine, arnottianamine, and oxynitidine) (Krane et al., 1984) and 4 furoquinoline alkaloids (β-fagarine, dictamnine, haplopine, and 3-(1,2-dihydroxyethyl)-4-methoxy-2(1H)-quinolinone) (Adamska-Szewczyk et al., 2016) (Supplementary Figure S2). The structures of the isolated alkaloids were established based on their NMR data and by comparison with those previously reported.

### The Commercially Available Alkaloid Nitidine Is a Powerful Synthetic Lethality-Inducer in BRCA1-Deficient Cells

**FIGURE 3:**
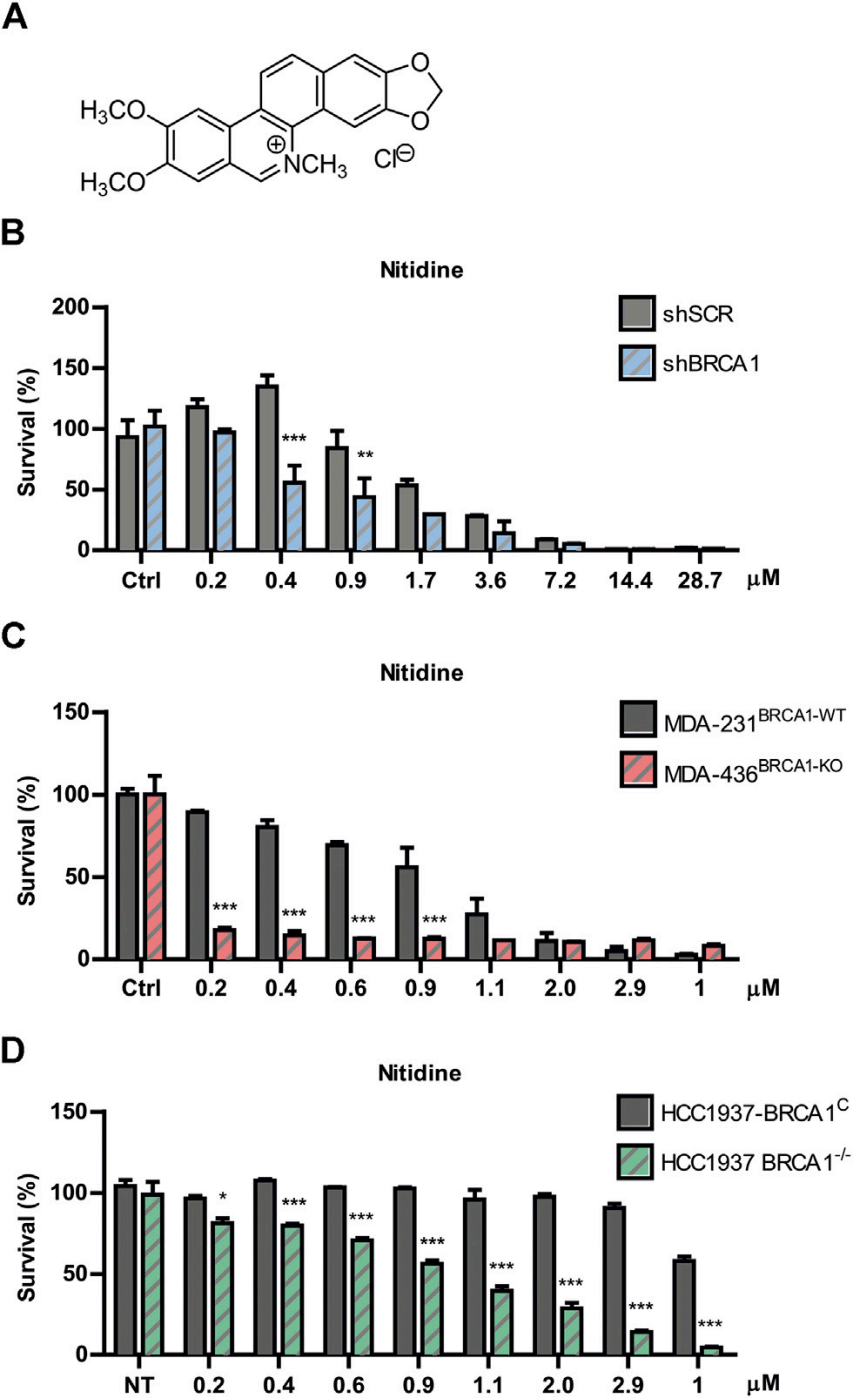
Evaluation of nitidine as a synthetic lethality (SL)-inducer in BRCA1-deficient cells. **(A)** Chemical structure of nitidine chloride. Determination of SL induction using BRCA-proficient and BRCA-deficient cells in a dose-response curve of nitidine after 6 days of treatment. The isogenic shBRCA1 and shSCR HCT116^p21−/−^ cells **(B)**; the triple-negative breast cancer MDA-MB-231^BRCA1-WT^ and MDA-MB-436^BRCA1-KO^ cells **(C)** and the isogenic breast cancer HCC1937 BRCA1^−/−^ and its complemented counterpart (HCC1937 BRCA1^C^) cell lines **(D)** were used. Statistical analysis was performed using two-way ANOVA with Bonferroni post-test (***, *p* ≤ 0.001; **, *p* ≤ 0.01; *, *p* ≤ 0.05).

Nitidine chloride (Figure 3A) is a bioactive benzophenanthridine alkaloid found in species of the *Zanthoxylum* genus, particularly in *Zanthoxylum nitidum* (Hu et al., 2006; Cai et al., 2007). It was described to have anti-inflammatory, anti-parasitic, and antifungal properties (Nhiem et al., 2020). Interestingly, this metabolite was reported to possess potent antitumor activities by inhibiting proliferation and inducing apoptosis in several types of cancer models (Chen et al., 2012; Liao et al., 2013; Fang et al., 2013; Cui et al., 2020; Nhiem et al., 2020). Since oxynitidine is the 6-oxo analogue of nitidine, we decided to test whether nitidine also depicts selective cytotoxicity against BRCA1-deficient cells. Surprisingly, nitidine exhibited strong SL-inducing activity, even in the nanomolar range, and it was presumably at least 10 times more potent than its 6-oxo analogue (Figure 3B). Presumably, this could be a consequence of the quaternary amino group present in nitidine, while oxynitidine has an amide functional group. The positive charge density of both alkaloids is very different, impacting in the interactions with the amino acids present in target molecules and in its pharmacokinetic properties.

To validate the SL phenotype in different genetic backgrounds, we performed survival experiments in two breast cancer models knockout (KO) for BRCA1. We used the non-isogenic pair of triple negative breast cancer MDA-MB cell lines (MDA-231^BRCA1-WT^ vs. MDA-436^BRCA1-KO^) and the isogenic HCC1937 model (BRCA1^KO^ vs. BRCA1^C^). We found that nitidine triggered SL in both BRCA1-KO backgrounds in a dose-response manner (Figures 3C,D). Noticeably, these two models presented higher SL sensitivity to nitidine and a wider dose-response range than the BRCA1-deficient cells generated by downregulation of BRCA1 using lentiviral shRNA (Figure 3B). Taken together, these data suggest that nitidine treatment is synthetic lethal with BRCA1 deficiency.

### AKT and Topoisomesase Inhibitors are Powerful Synthetic Lethality-Inducers in BRCA1-Deficient Cells

**FIGURE 4:**
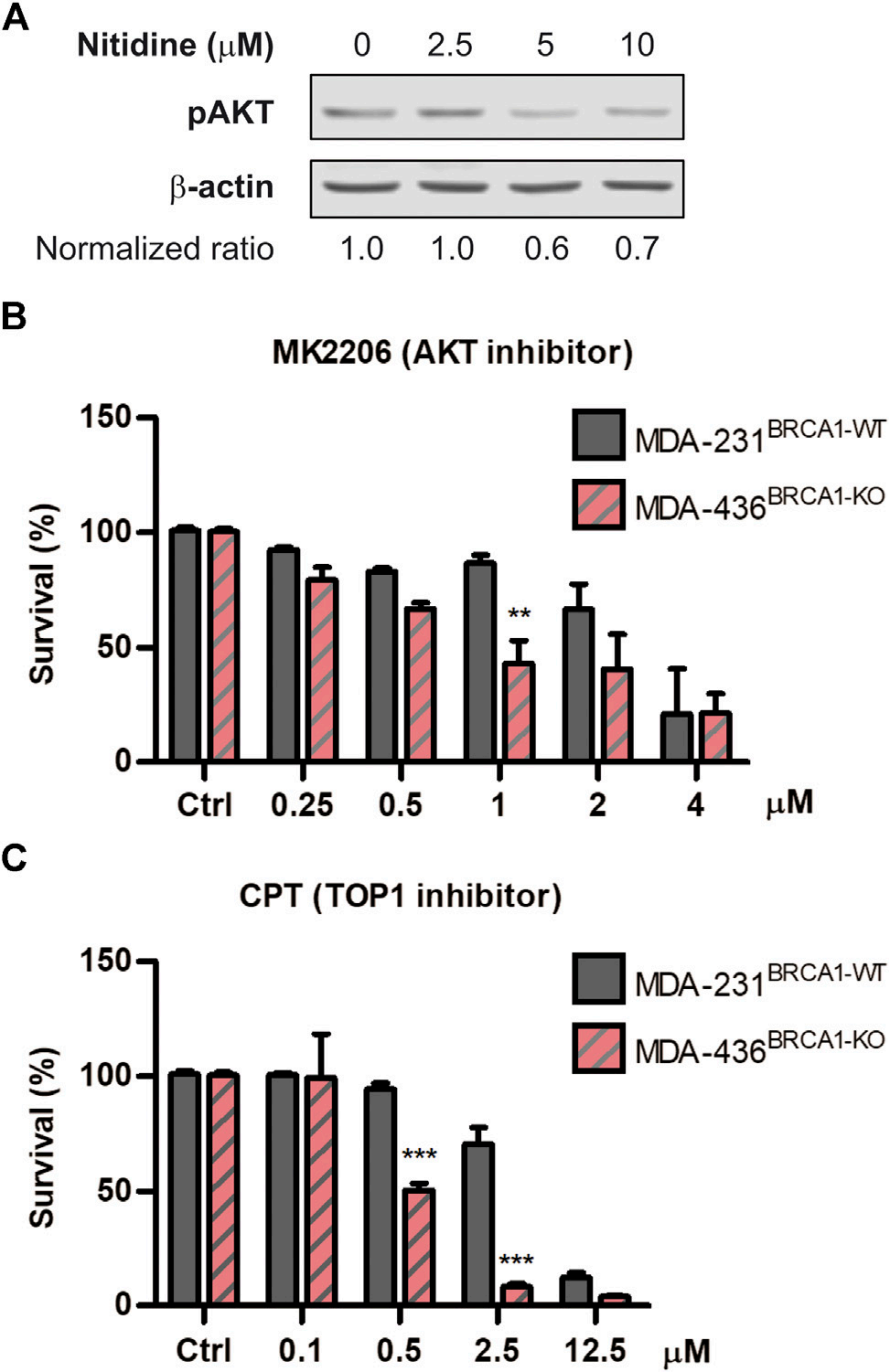
Analysis of the cytotoxic effect of AKT and Topoisomerase I inhibition in BRCA-deficient cells. **(A)** western blot confirming the efficient impairment of AKT phosphorylation after the treatment with different doses of nitidine (0, 2.5, 5 and 10 µM) in breast cancer cell lines MDA-MB-236 for 3 h. β-Actin was used as loading control. The normalized *p*-AKT/β-Actin ratios are shown. Determination of synthetic lethality induction in MDA-MB-231^BRCA1-WT^ and MDA-MB-436^BRCA1-KO^ cells after 6 days of treatment with AKT inhibitor, MK2206 **(B)**, or Topoisomerase I inhibitor, Camptothecin **(C)** in a dose-response range. Statistical analysis was performed using two-way ANOVA with Bonferroni post-test (***, *p* ≤ 0.001; **, *p* ≤ 0.01; *, *p* ≤ 0.05).

To explore the underlying mechanism of action associated to the SL-induction triggered by nitidine, we explored different hypotheses. While several biological properties were reported for nitidine (Reviewed in Nhiem et al., 2020), we focused on two main activities that might be responsible for its selective antitumoral effect in BRCA1-deficient contexts. On the one hand, it was described that nitidine disrupts AKT signaling (Fang et al., 2013; Cheng et al., 2016; Ding et al., 2016), which was recently reported to be involved in SL-induction in BRCA1-deficient cells (Villafañez et al., 2019). On the other hand, nitidine inhibits Topoisomerase I and Topoisomerase II activities (Fang et al., 1993; Wang et al., 1993; Holden et al., 1999; Liu et al., 2018), which were also reported to trigger SL in BRCA1^−/−^ cellular models (Maede et al., 2013). We initially confirmed that nitidine inhibits AKT signaling using our experimental settings by analyzing phospho-AKT levels in breast cancer cell line MDA-MB231 (Figure 4A). Then, we evaluated the cytotoxic effect of AKT inhibitor MK2206 (Figure 4B) and the Topoisomerase I inhibitor Camptothecin (Figure 4C) in BRCA1-deficient cells. Our results revealed that both inhibitors induced SL in BRCA1-KO cells (Figures 4B,C). Taking together, our findings indicate that AKT and topoisomerases are the potential molecular targets responsible of nitidine-triggered SL-induction in BRCA1-deficient cells.

## Discussion

Cancer is responsible for millions of deaths worldwide every year (Torre et al., 2016) (https://gco.iarc.fr/). Carcinogenesis is a multi-step process at which several mechanisms that control cell division sequentially fail, leading to transformed phenotypes with unrestricted cell proliferation and survival (Hann and Weinberg 2007; Puisieux et al., 2018). In such a complex disease, redundancies and complexities of biological pathways often lead to compensation, treatment failure and resistance to targeted therapies (Ramsay et al., 2018). Moreover, single-target therapeutic approaches may be affected by side effects and tissue toxicity, resulting in reduced efficacy and a decreased quality of life for patients (Anighoro et al., 2014). In contrast, specific drug combinations or multi-targeted therapy are designed to achieve more durable disease control, resulting from simultaneous blockade of disease-relevant targets (Anighoro et al., 2014). Moreover, it was also suggested that molecules with multitarget mechanism of action may possess a safer profile compared to single-target ones (Bolognesi, 2013; Ramsay et al., 2018). Therefore, successful treatments for cancer may require targeting multiple disease-causing pathways to achieve additive or synergistic effects, as well as overcoming drug resistance. In consequence, many novel drug discovery and development strategies are being focused on targeting multiple molecular pathways, either with drug combinations or through the design of single compounds that target multiple proteins: an approach known as polypharmacology (Anighoro et al., 2014; Ho et al., 2018; Lim et al., 2019).

One of the key advantages of using natural products instead of synthetic compounds in drug discovery is that natural products are likely to be multi-target compounds. Nitidine is a common alkaloid found in many *Zanthoxylum* species, and it has been reported to suppress cell proliferation, to stimulate apoptosis, to induce cell cycle arrest, and to retard migration, invasion and metastasis in a variety of malignancies via multiple molecular mechanisms (Cui et al., 2020; Nhiem et al., 2020). In this work, we unveil a potential polypharmacological capacity for nitidine, which might be responsible for its synthetic lethal activity against BRCA1-deficient cells. Our working model is that the selective and robust antitumoral activity of nitidine treatment is a consequence of the simultaneous targeting of two molecular pathways of central relevance in BRCA1^−/−^ models: AKT and topoisomerases (Maede et al., 2013; Villafañez et al., 2019). Interestingly, while these studies shown that the inhibition of such targets are linked to the impaired viability of BRCA1-deficient cells *in vitro*, these types of approaches are not yet established as alternatives for the treatment of BRCA1-related cancers. However, a couple of recent reports shed light on this direction. First, it was shown that AKT inhibition using the investigational drug MK-2206 suppresses the initiation and progression of BRCA1-associated mammary tumors (Baek et al., 2018). Second, a recent report indicates that BRCA deficiencies predict response to topoisomerase I inhibitors in 40 PDXs obtained from triple-negative breast cancers patients (Coussy et al., 2020).

The findings in this work also highlight the importance of exploring alkaloids from the *Zanthoxylum* genus. This is one of the biggest genera belonging to the Rutaceae family, which includes 212 species widely distributed in areas with warm or tropical climate. Many biological activities were described in *Zanthoxylum* spp., such as antimicrobial, anti-inflammatory, antioxidant, and in particular cytotoxic activity. Even though several classes of compounds have been isolated from this genus, alkaloids are the components with the most interesting biological properties (Nhiem et al., 2020). Particularly, this genus holds elevated levels of benzophenanthridine alkaloids that not only revealed their potential cytotoxic *in vitro* but also their capability to inhibit tumor growth *in vivo* through several mechanisms (Nhiem et al., 2020). Furoquinoline alkaloids are also found in *Zanthoxylum* species, and they show cytotoxic activity as well (Yang et al., 2009; Nhiem et al., 2020). The findings of this work unveil an intriguing new aspect of the alkaloids from *Zanthoxylum-*spp., positioning them as promising lead molecules for future drug discovery projects.

## Data Availability Statement

The raw data supporting the conclusions of this article will be made available by the authors, without undue reservation, to any qualified researcher.

## Author Contributions

Conceptualization, IG, MP, MC, VN, and GS; methodology, IG, MP, AP, MEG, and MLG; acquisition of data: MP, MCC, JB, GB, and JO; investigation, IG, MP, and MEG; writing-original draft, IG and GS; supervision, JB, MC, VN, and GS; project Administration, GS; funding acquisition: GS. All authors contributed to manuscript revision, read and approved the submitted version.

## Funding

This work was supported by a consortium grant from FONCyT and the Trust in Science program (Global Health R&D) from GlaxoSmithKline (PAE-GLAXO 2014-0005). Additional support came from grants: PICT 2015-0543, PICT 2016-0235 from FONCyT and from National Institute of Cancer of Argentina (INC-2018). IG and MG were supported by fellowships from PAE-GLAXO 2014-0005. MFP was supported by fellowships from the National Institute of Cancer and CONICET. MG, JO, JB, MC, GB, VN, and GS are researchers from CONICET. AP is researcher from UNC. The authors declare that this study received funding from the Trust in Science Program of GlaxoSmithKline. The funder was not involved in the study design, collection, analysis, interpretation of data, the writing of this article or the decision to submit it for publication.

## Conflict of Interest

The authors declare that the research was conducted in the absence of any commercial or financial relationships that could be construed as a potential conflict of interest.
