# Supplementary material for: Synthetic Lethal Activity of Benzophenanthridine Alkaloids From Zanthoxylum coco Against BRCA1-Deficient Cancer Cells
Source: Front Pharmacol. 2020 Dec 3;11:593845. doi: 10.3389/fphar.2020.593845 (PMC7793782; doi:10.3389/fphar.2020.593845)
Supplement: Supplementary file 3 [file datasheet1.pdf]

Supplementary Material

A

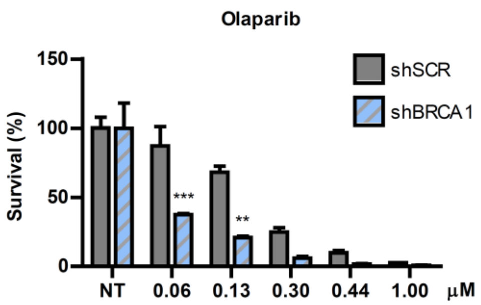

B

Zanthoxylum coco (HCT116$^{p21-/-}$)				
Extract	Extraction	µg/mL	Surv. Dif.	SL Fold
Fruits	Alkaline	33.3	0.69	0.27
Fruits	Alkaline	13.3	47.87	0.90
Fruits	Alkaline	5.3	1.77	0.16
Fruits	Alkaline	2.1	2.17	0.02
Fruits	Acid	33.3	2.54	0.98
Fruits	Acid	13.3	2.27	0.27
Fruits	Acid	5.3	4.70	0.11
Fruits	Acid	2.1	0.36	0.06
Leaves	Alkaline	33.3	1.03	0.44
Leaves	Alkaline	13.3	2.21	0.29
Leaves	Alkaline	5.3	0.00	0.06
Leaves	Alkaline	2.1	0.00	0.10
Leaves	Acid	33.3	0.21	0.08
Leaves	Acid	13.3	1.92	0.60
Leaves	Acid	5.3	1.32	0.15
Leaves	Acid	2.1	7.23	0.06

Supplementary Figure 1. A. Positive control to calibrate the robustness of the SL induction assay at 6 days using the PARP inhibitor Olaparib in dose-response range, which is selectively toxic against the BRCA-deficient population. Statistical analysis was performed using two-way ANOVA with Bonferroni post-test (***, $P \leq 0.001$; **, $P \leq 0.01$). **B.** Results obtained in dose-response experiments testing different extracts obtained from Z. coco in HCT116$^{p21-/-}$ cell lines. The SL fold and the survival difference parameters are detailed.

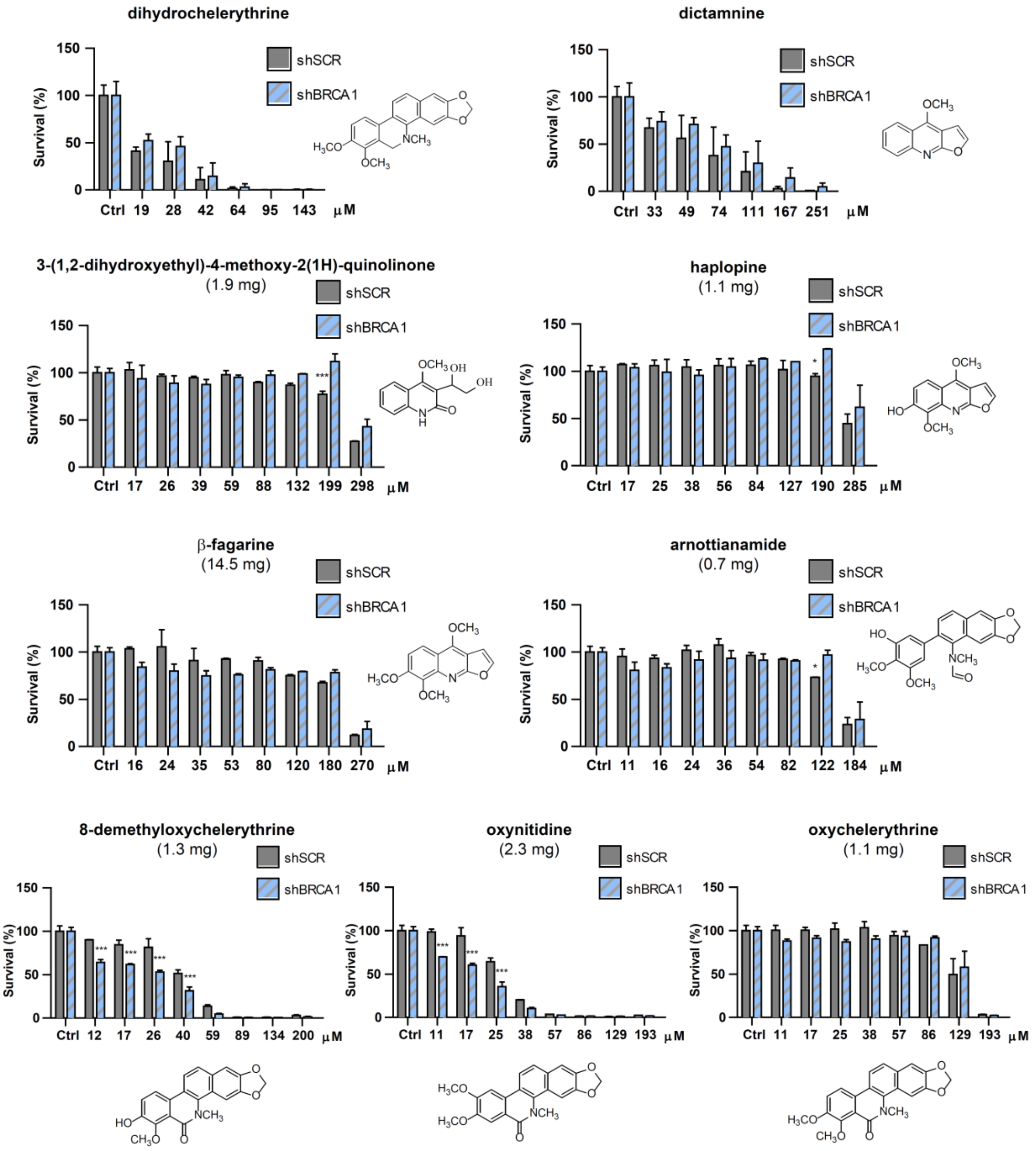

Supplementary Figure 2. Determination of SL induction using HCT116$^{p21-/-}$ BRCA-proficient and BRCA-deficient cells in a dose-response curve of the 9 alkaloids isolated and identified from *Z. coco* active extract. Their chemical structures are shown. Statistical analysis was performed using two-way ANOVA with Bonferroni post-test (***, $P \leq 0.001$; **, $P \leq 0.01$; *, $P \leq 0.05$).
